# Frozen State of Sephadex^®^ Gels of Different Crosslink Density Analyzed by X-ray Computed Tomography and X-ray Diffraction

**DOI:** 10.3390/gels4020044

**Published:** 2018-05-18

**Authors:** Norio Murase, Yuki Uetake, Yuki Sato, Kentaro Irie, Yohei Ueno, Toru Hirauchi, Toshio Kawahara, Mitsuhiro Hirai

**Affiliations:** 1 Division of Life Science and Engineering, School of Science and Engineering, Tokyo Denki University, Hatoyama, Hiki-gun, Saitama 350-0394, Japan; uetake.yuki0414@gmail.com (Y.U.); yuuki_satou01@yahoo.co.jp (Y.S.); 2 Nisshin Seifun Group Inc., Research Center for Basic Science, Research and Development, Quality Assurance Division, 5-3-1 Tsurugaoka, Fujimino-city, Saitama 356-8511, Japan; irie.kentaro@nisshin.com (K.I.); ueno.yohei@nisshin.com (Y.U.); hirauchi.toru@nisshin.com (T.H.); kawahara.toshio@nisshin.com(T.K.); 3 Graduate School of Science and Technology, Gunma University, 4-2 Aramaki, Maebashi, Gunma 371-8510, Japan; mhirai@gunma-u.ac.jp

**Keywords:** Sephadex^®^ (crosslinked dextran), crosslink density (density of crosslinks), ice grain, ice crystallization during rewarming, glassy water, X-ray CT, XRD

## Abstract

Water in Sephadex^®^ (crosslinked dextran) gels is known to indicate different freezing behavior which is dependent on the density of the crosslinks, and water in a Sephadex^®^ G25 gel remains partially unfrozen during cooling and crystallizes during rewarming. The mechanism of anomalous ice crystallization during rewarming is still unclear. The objective of this study is to observe the ice grains that form in Sephadex^®^ beads and to comprehend their frozen state with a focus on the ice crystallization during rewarming. Sephadex^®^ beads containing 50 wt % water were prepared and used for the measurements. The observation of the ice grains was carried out by using synchrotron radiation-sourced X-ray CT (computed tomography). XRD (X-ray diffraction) analysis was also conducted to investigate the frozen state. As a result, ice grains that were larger than ~1 μm were hardly observed after the slow cooling of Sephadex^®^ beads, except in the G25 beads. However, at the occurrence of ice crystallization during rewarming, ice grains that were larger than 10 μm appeared in the G25 beads. Using XRD, it was found that small incomplete ice crystals were formed in G25 beads and the presence of glassy water was indicated in the gel. In conclusion, the size and distribution of ice grains that formed in Sephadex^®^ beads were different depending on the density of the crosslinks.

## 1. Introduction

Biological systems often take a gelled state. Understanding the frozen state of gels (i.e., the size and distribution of ice grains) is of practical importance for the implementation of cryopreservation. Water in gels is compartmentalized by the polymer network [1,2], and there is a possibility that the polymer network obstructs and retards the diffusional motion of the water molecules in gels [3], consequently preventing the growth of ice crystals. The study of the freezing behavior and the state of the frozen gels is of basic interest, as they reflect the characteristics of the polymer network (i.e., the flexibility of polymer chains, the size of the compartments, and the extent of the continuity between adjacent compartments which are interrelated via the density of the crosslinks) [4].

In this connection, the freezing behavior of Sephadex^®^ (crosslinked dextran) gels has been investigated mainly by DSC (differential scanning calorimetry) [1,2] and by various physical techniques [5]. Stemming from these investigations, different freezing behaviors were indicated depending on the density of the crosslinks. It was found that with the Sephadex^®^ G25 gel, the water remains partially unfrozen during cooling and the ice crystallizes during the subsequent warming (rewarming), as indicated by DSC. A conceivable scheme of ice crystallization during rewarming is described as follows. Some part of the water in the gel is trapped by the polymer network at the time of freezing initiation when it is cooled, followed by a change in the polymer network that turns it into a glassy state. The glassy water crystallizes during the rewarming, caused by the partial melting of the ice that was previously separated [5,6,7]. The presence of glassy water, however, remains unconfirmed. Even when the water does not turn into a glassy state, small ice crystals are formed in the gel and a powder diffraction pattern has been observed by a two-dimensional X-ray diffraction (XRD) study [8]. In Sephadex^®^ gels with crosslinks of both higher and lower densities than that of G25, an anomalous freezing behavior during rewarming was not observed, and the larger ice crystals were considered to have formed, which was also indicated by the two-dimensional XRD study [8].

As the glass transition temperature (*T*_g_) of hyper-quenched liquid water is known to be around 136 K (−137 °C) [9], the *T*_g_ of water in a G25 gel (assumed to be around −50 °C) is too high. The *T*_g_ of the water in the G25 gel was estimated from the temperature where the liquid water disappears in the ESR (Electron Spin Resonance) spectra obtained by using a spin probe method. The water in the G25 gel is partially trapped by the polymer network and turns into a glassy state at the time of freezing initiation, presumably as a consequence of the close interaction with the hydroxy groups located at the surface of the dextran network structures via the hydrogen bonds. The high *T*_g_ value of the water in the gel can thus be explained. In fact, the *T*_g_ of bulk water confined in MCM-41 nanopores was suggested to be 210 K [10].

The occurrence of ice crystallization during rewarming observed with Sephadex^®^ G25 gel is characteristic of non-equilibrium freezing after substantial supercooling, and it is not observed after equilibrium freezing [7]. In this connection, supercooled water is of great interest from a scientific standpoint and has been actively discussed since the 1970s [11]. Although various thermodynamic properties such as the heat capacity (*C*_p_), the isothermal compressibility, and so forth have indicated a divergence at around −45 °C, the behavior of said divergence is a matter of concern [11,12] and it is still unclear due to the interference by homogeneous nucleation which occurs at around −40 °C. Concerning the amorphous phases of ice, the high-density and low-density amorphous forms of ice and/or polymorphism—where a deeper understanding of the hydrogen bond network in water is required [13]—have also been intensively discussed. Although the presence of supercooling is a requisite for the observation of ice crystallization during rewarming, the water in the gel freezes at around −22 °C; the degree of supercooling is not very high. Then, the rates of freezing and the resultant change of the polymer network dependent on the degree of supercooling need to be fast for the observation and the occurrence of the glass transition. The analysis of ice crystallization during rewarming observed with a G25 gel might provide valuable information in this connection.

Ice crystals exist in aggregates as ice grains, and small ice grains are formed as a consequence of the formation of small ice crystals. Then, the sizes of the ice grains observed reflect the sizes of ice crystals that are formed. Although it might be true that the size of ice crystals (and therefore the ice grains formed) are different depending on the density of the crosslinks or on the polymer network structure, the information obtained by the diffraction pattern of the two-dimensional XRD is unsatisfactory, as there is no information on the distribution of the ice grains in the gels. The direct observation of ice grains at a high resolution is desirable for the understanding of the frozen state. The observation of the beads by SEM (scanning electron microscope) after freeze-drying is possible. However, there is concern that the technique will lead to changes in the polymer network during the freeze-drying treatment.

In the present study, the in situ observation of the ice grains formed in Sephadex^®^ gel beads was conducted by the X-ray CT (computed tomography) imaging technique. Recently, the X-ray CT technique has been improved, making it possible to acquire three-dimensional images of the microstructure of foods or biological systems [14,15]. The technique of X-ray CT imaging is based on the detection of the differences in the X-ray absorption rate by the materials that compose the sample. The contrast of the images depends on the wavelength, chromaticity (mono- or poly-), and the brilliance of the X-ray beam applied, as well as the mass density and chemical components of materials. By using synchrotron radiation, a highly brilliant and monochromatic X-ray beam can be provided and a high spatial resolution was made possible. Then, the synchrotron radiation-sourced X-ray CT analysis of the ice grains formed in Sephadex^®^ beads was conducted at SPring-8, a synchrotron radiation facility in Japan. The detection of ice grains in the micrometer range by using a beam line in the facility was anticipated. The observation of the freeze-dried Sephadex^®^ beads by SEM was also conducted for comparison.

To understand the frozen state of gels, it is necessary to obtain information about the ordered structure of the polymer network forming the gel as well as the size of the ice grains that are smaller than a micrometer. For that purpose, synchrotron radiation-sourced SAXS (small-angle X-ray scattering) measurements were also carried out at SPring-8. For the analysis of the completeness of ice crystals determining the shape of the ice grains, WAXD (wide-angle X-ray diffraction) measurements were carried out together with SAXS measurements in the XRD study. As the XRD study deals with the structure of dimensions that are smaller than the beads, the term “gel” was used for the XRD experiment instead of “beads.”

The objective of the present study was to analyze the shape and distribution of the ice grains formed in the Sephadex^®^ beads with different crosslink densities, as well as to comprehend the frozen state, especially that of the G25 gel, that leads to ice crystallization during rewarming.

## 2. Results

### 2.1. X-ray CT Measurement

**Figure 1:**
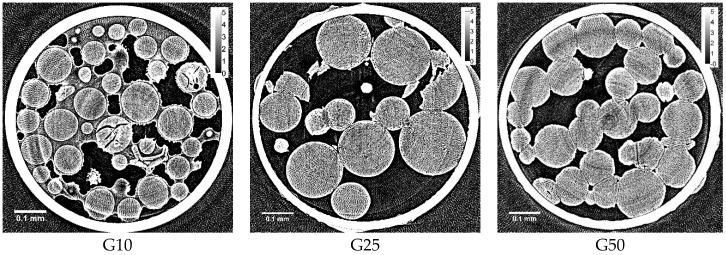
X-ray computed tomography (CT) images obtained with rapidly cooled Sephadex^®^ beads containing 50 wt % water: (**left**) G10; (**middle**) G25; (**right**) G50 beads. Small white circles different from the beads observed in G25 and G50 beads might be artifacts during the measurement. Calibration bars on the upper right indicate the linear X-ray absorption coefficient (LAC, cm^−1^). The density of the crosslinks: G10 > G15 > G25 > G50 > G100 [16].

Typical X-ray CT images obtained with Sephadex^®^ beads after rapid cooling are shown in Figure 1. Linear X-ray absorption coefficients (LACs) obtained for ice and the bead matrix in the figure were about 2.2 and 3.0, respectively. The LAC order was quartz capillary > bead matrix > ice > air, and the bead matrix observed in the capillary is represented as gray circles. The ice grains are represented in a gray color slightly darker than the bead matrix.

In the case of G10 beads, ice due to unabsorbed water was seen around the beads. Slits observed dark in some beads were due to the cracks formed during freezing. However, spots due to the ice grains were not identified in the internal region of G10 beads, indicating that ice grains smaller than ~1 μm were formed in the beads. In the internal region of G25 and G50 beads, the ice grains were not identified either. Several fragments of the beads observed in the image of G25 beads were probably made by breakage during the sample packing into the capillary.

**Figure 2:**
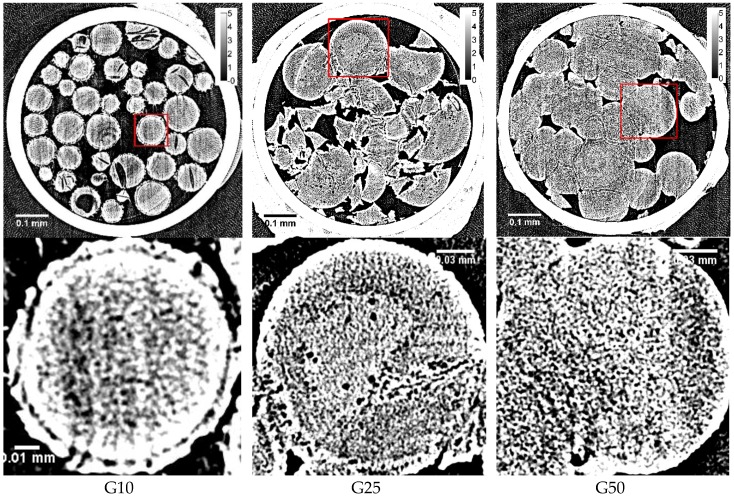
X-ray CT images obtained with slowly cooled Sephadex^®^ beads containing 50 wt % water (**top**) together with the expanded images of the square part of the corresponding upper ones (**bottom**): (**left**) G10; (**middle**) G25; (**right**) G50. Calibration bars on the upper right indicate the LAC (cm^−1^).

Typical X-ray CT images obtained with the beads after slow cooling are shown in Figure 2.

Among the three kinds of Sephadex^®^ beads, there was a difference in the appearance of the surface as well as in the degree of the connection between adjacent beads. G10 beads were isolated from each other. On the other hand, adjacent G50 beads were ready to stick together. The degree of stickiness of G25 beads resided between G10 and G50 beads. Large spots observed on the margin of G10 beads were probably due to the ice grains. With G25 beads, large spots were also observed on the margin of the beads. However, no large ice grains were observed on the margin of G50 beads. 

The dark spots of 3‒5 μm in diameter observed in the internal region of G25 beads were due to the ice grains, though they are not very abundant. With G10 and G50 beads, spots due to the ice grains larger than ~1 μm were not identified in the internal region.

**Figure 3:**
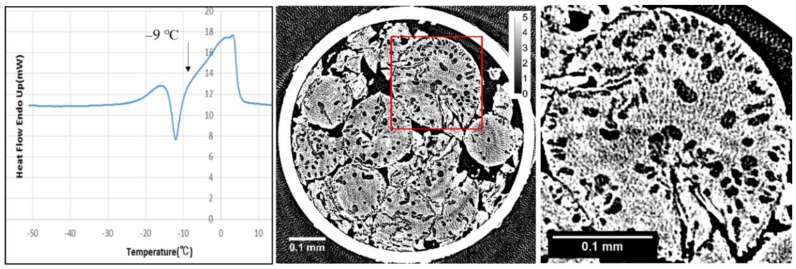
X-ray CT images obtained with refrozen Sephadex^®^ G25 beads re-cooled from −9 °C (**middle**) together with the expanded image of the square part (**right**), and a differential scanning calorimetry (DSC) rewarming trace indicating the ice crystallization exotherm (**left**). DSC cooling and heating rates were 5 °C min^−1^.

**Figure 4:**
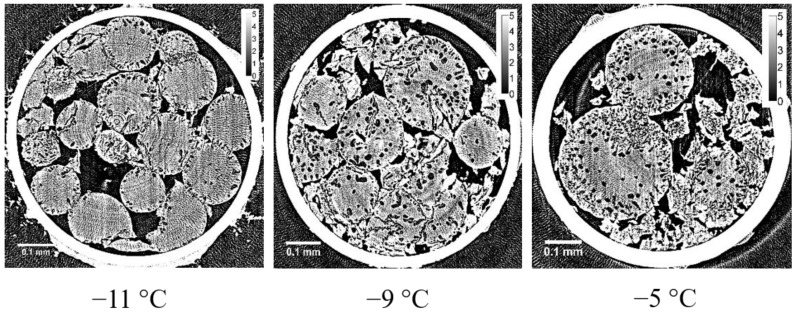
Dependence of X-ray CT images of refrozen G25 beads on the temperature from which the re-cooling was initiated: (**left**) −11 °C; (**middle**) −9 °C; (**right**) −5 °C.

X-ray CT images obtained with refrozen G25 beads re-cooled from −9 °C (the temperature of the completion of the ice crystallization exotherm) are shown in Figure 3 together with the DSC heating trace. Large ice grains larger than 10 μm appeared after the occurrence of ice crystallization during rewarming. The dependence of the X-ray CT images of refrozen G25 beads on the temperature from which re-cooling was initiated is shown in Figure 4. When the re-cooling was initiated from −9 °C, large ice grains observed in the X-ray CT image were most abundant among the images different in the initiation temperature of re-cooling, −11 or −5 °C.

### 2.2. Observation by SEM

**Figure 5:**
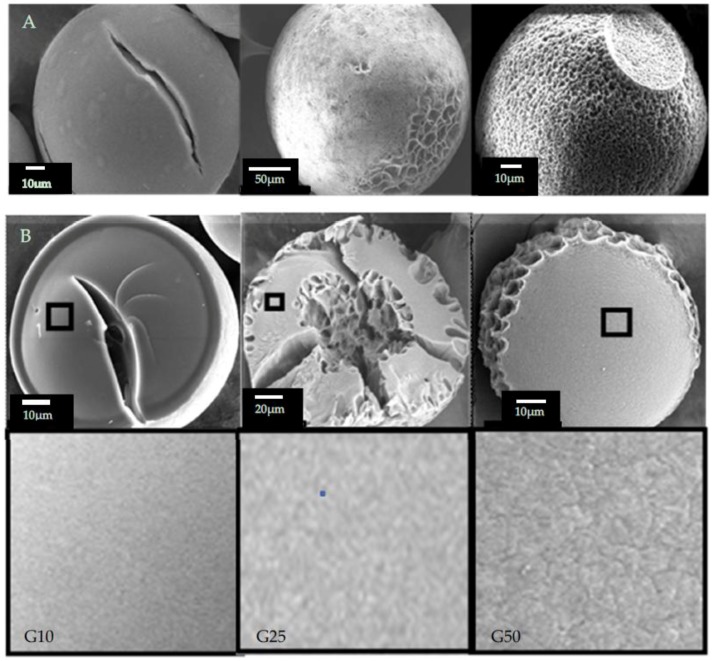
SEM images of Sephadex^®^ beads after freeze-drying. (**A**) Beads; (**B**) Surface of the fractured beads (**top**) and the expanded images of the square part of the corresponding upper ones (**bottom**): (**left**) G10; (**middle**) G25; (**right**) G50.

Sephadex^®^ beads observed by SEM after freeze-drying are shown in Figure 5. In the G10 bead, a crack was clearly observed. The surface of the bead appeared to be smooth and rigid. With the G25 bead, hollows indicating the trace of the ice grains were observed in the internal region of the beads as well as on the margin. These characteristics of the SEM images correspond to those obtained by X-ray CT. However, clear difference in the network structure of the bead matrix was not confirmed among the Sephadex^®^ beads used.

### 2.3. XRD Measurement

**Figure 6:**
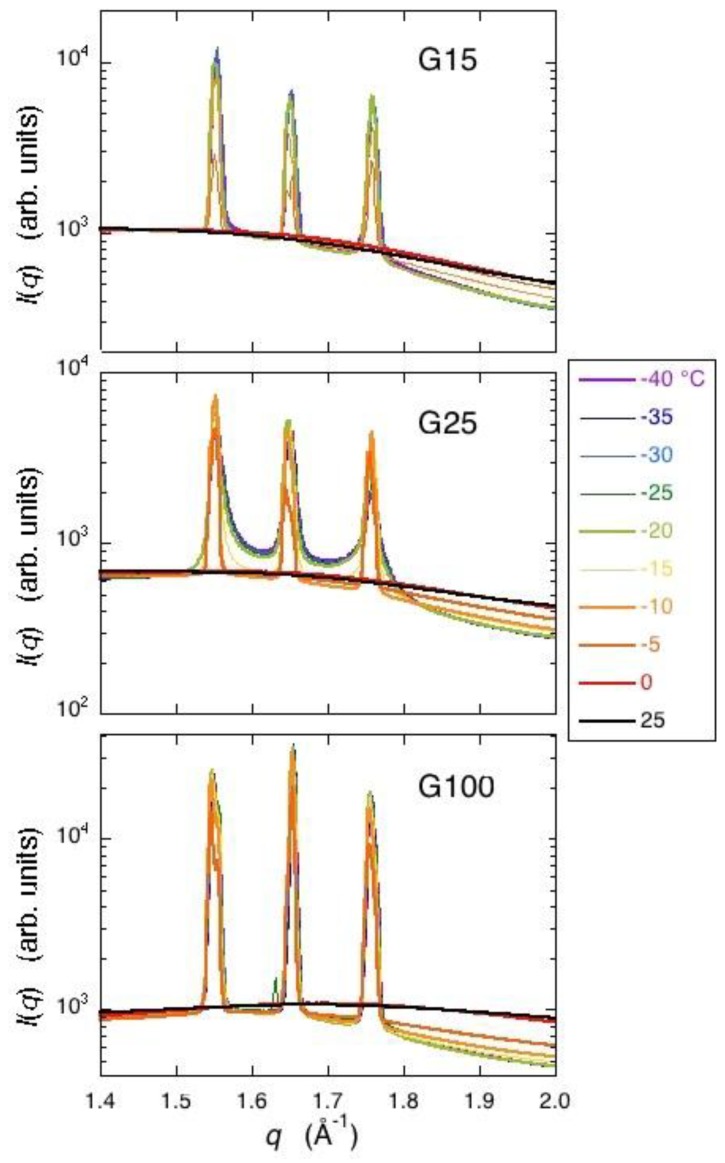
Temperature dependence of the WAXD (wide-angle X-ray diffraction) curves obtained with frozen Sephadex^®^ gels. G15, G25, and G100 gels from the top to the bottom, respectively.

The SAXS measurement was carried out to obtain information about the structure of the polymer network or the size of the ice grains smaller than the micrometer order. However, characteristics dependent on the density of crosslinks were not confirmed with the frozen Sephadex^®^ gels. On the other hand, in the WAXD measurement, Bragg peaks due to the hexagonal ice observed at around 0.155‒0.175 for the scattering vector *q* (10 × nm^−1^) indicated the different temperature dependence among the three Sephadex^®^ gels, G15, G25, and G100, as shown in Figure 6.

**Figure 7:**
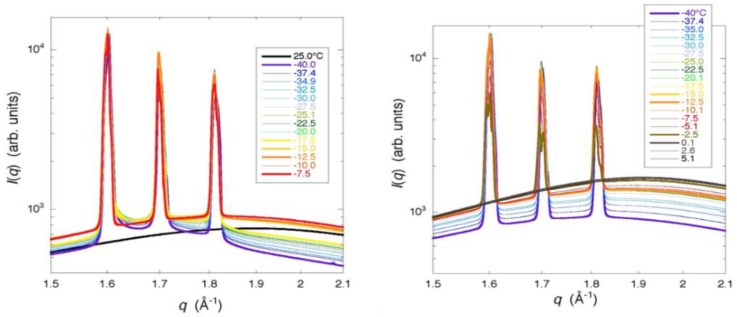
Temperature dependence of the WAXD curves obtained with frozen and refrozen G25 gels. (**left**) Bragg peaks observed with a frozen G25 gel; (**right**) Bragg peaks observed with a refrozen G25 gel re-cooled from −7.5 °C. The temperature interval in this experiment was 2.5 °C.

Bragg peaks of a frozen G25 gel observed at −40 °C showed a remarkable tailing suggesting the formation of small incomplete ice crystals or the presence of glassy water. With the increase in temperature, the peaks became narrow at temperatures between −20 and −15 °C, where an ice crystallization exotherm during rewarming was initiated in the DSC trace. The narrowing trend continued to −5 °C and the peaks disappeared at temperatures between −5 and 0 °C, where ice melted. Bragg peaks observed with a frozen G25 gel depending on the freezing condition are shown in Figure 7. When the heating of the frozen G25 gel was interrupted at the temperature of the completion of ice crystallization during rewarming followed by re-cooling, Bragg peaks of the refrozen G25 gel became narrow without tailing through the temperature from −40 °C to the melting temperature, suggesting the formation of large stable ice crystals at the occurrence of crystallization.

Bragg peaks of a frozen G15 gel observed at −40 °C also showed a slight tailing (Figure 6), which weakened with the increase in temperature. On the other hand, in the case of a frozen G100 gel, narrow Bragg peaks were observed through temperatures ranging from −40 °C to the melting temperature, indicating the formation of large stable ice crystals during cooling.

## 3. Discussion

By the present study of X-ray CT measurement using synchrotron radiation, the in situ observation of the ice grains in Sephadex^®^ beads was made possible with a spatial resolution of ~1 μm. As a result, it is necessary to change the interpretation of the size and distribution of the ice grains formed in the beads that was held previously.

By the previous study of the two-dimensional XRD measurement, it was interpreted that ice crystals of larger size were formed in a G10 gel compared to those in G25 and G100 gels, as many clear spots were observed on the three concentric rings due to the diffractions by hexagonal ice in the image [8]. Moreover, it was considered that the formation of large ice crystals corresponded to the formation of the large ice grains in the internal region of G10 beads. However, clear spots observed by the two-dimensional XRD measurement were probably due to the ice crystallized outside the G10 beads as the unabsorbed water existed, which was indicated by the X-ray CT image obtained after rapid cooling (Figure 1). Although the water holding capacity of a G10 gel is 1 g water/g dry gel, corresponding to a water content of 50 wt % according to the literature [16], it is reasonable to consider that some water remains unabsorbed outside the gel beads because of the inhomogeneous distribution of water in the bead sample. There is another possibility of the observation of clear spots in the two-dimensional XRD image of a G10 gel—the large ice grains may be formed in the region of the beads between the rigid surface-treated layer and the polymer matrix. The existence of this region is suggested by the image in Figure 2. However, no ice grains larger than ~1 μm were observed in the internal region of G10 beads, even after slow cooling, though most of the water in the beads was frozen. By the DSC measurement, about 70 percent of the water in the Sephadex^®^ beads was estimated to be frozen during slow cooling [2]. Looking at the expanded X-ray CT images of the beads (Figure 2), small structures of ~1 μm seemed to appear in the internal region of the beads. However, they might be artifacts, as the fluctuation of the X-ray absorption rate is considerable, especially at the border between ice and the polymer matrix. Ring artifacts were also observed, as can especially be seen in Figure 1.

In the internal region of G25 beads, a small number of ice grains of 3‒5 μm in diameter were observed after slow cooling. Although the ice grains were observed in G25 beads, most of the ice grains that formed in the rest of the beads were smaller than ~1 μm, as they were not visible in the image. This is consistent with the results obtained from the two-dimensional XRD study where the powder diffraction pattern was observed [8]. In the X-ray CT image obtained with the refrozen G25 beads re-cooled from −9 °C (the temperature of the completion of ice crystallization during rewarming), many large ice grains larger than 10 μm appeared in the internal region of the beads, which is also consistent with the result of the two-dimensional XRD study where the spotty diffraction pattern indicating the formation of large ice crystals was observed.

There were no surface-treated layers observed in G50 beads. Instead, G50 beads containing 50 wt % water were ready to stick together, probably by means of the tangling bonds of the polymer chains existing on the bead surface. In this connection, G100 beads with lower crosslink densities than G50 beads became too sticky, losing the bead structure when they were added with water [17], making it difficult to prepare G100 bead samples in quartz capillaries. That is the reason why G50 beads were used in this study instead of G100 beads as the bead sample with low crosslink densities. No ice grains were observed in the CT image of G50 beads. Considering the result obtained by the two-dimensional XRD study using a G100 gel, where pairs of arcs facing each other were seen on the three concentric rings due to the diffractions by hexagonal ice [8], there is a possibility that long and narrow structures could be observed in the X-ray CT image of G50 beads. However, the width of the structures may have been too narrow (<~1 μm), if they existed, to be recognized by the observation.

From the results of the WAXD measurement, the formation of small incomplete ice crystals in a G25 gel was indicated, as Bragg peaks showed a remarkable tailing. The result is consistent with that of the two-dimensional XRD study where continuous but dim images due to the diffractions by powder crystals was obtained for hexagonal ice. Moreover, the presence of glassy water in a frozen G25 gel was indicated, though not decisive [5,18]. A slight tailing in the Bragg peaks was also observed with a frozen G15 gel, indicating the presence of small ice crystals in the gel with higher crosslink densities than a G25 gel. The finding is consistent with the interpretation of the result obtained with a G10 gel by the X-ray CT measurement. In the case of a G100 gel with lower crosslink densities than a G25 gel, the formation of large and stable ice crystals was indicated by WAXD, which is also consistent with the result obtained by the two-dimensional X-ray measurement, though the presence of long and narrow ice structures was not verified by the X-ray CT measurement as mentioned above. The observation of narrow Bragg peaks with a refrozen G25 gel indicated large and stable ice crystals. Therefore, large ice grains were formed in the beads at the time of ice crystallization during rewarming. This result corresponds with that of the X-ray CT measurement.

It is certain that the frozen state of the Sephadex^®^ gels depending on the density of the crosslinks was made clearer by the experiments of a synchrotron radiation-sourced X-ray CT and an XRD. However, the frozen state of the gels of submicron order remains unclarified. More precise SAXS measurements are necessary. Concerning ice crystallization during rewarming, the presence of glassy water in a G25 gel is still unconfirmed. Detection of the glass transition in the frozen gel is desirable for this clarification [10].

## 4. Conclusions

The frozen state of Sephadex^®^ gels containing 50 wt % water was analyzed by using a synchrotron radiation-sourced X-ray CT and XRD.

By X-ray CT, the ice grains in Sephadex^®^ beads were observed with a spatial resolution of ~1 μm. As a result, ice grains larger than ~1 μm were hardly observed in the beads after slow cooling, independent of the crosslink density. In G25 beads, a small number of ice grains of 3‒5 μm in diameter were observed after slow cooling, but most of the ice grains formed in the beads were smaller than ~1 μm. Ice grains larger than 10 μm in diameter were successfully observed in the refrozen G25 beads re-cooled from the temperature of the completion of ice crystallization during rewarming. These results obtained by the X-ray CT measurement were consistent with those previously obtained by two-dimensional XRD measurement. 

The results obtained by XRD—especially by WAXD—corresponded well with those obtained by X-ray CT. The observation of a remarkable tailing in Bragg peaks due to the diffraction by hexagonal ice indicated the formation of small incomplete ice crystals or the presence of glassy water in a G25 gel. With the refrozen G25 gel re-cooled from the temperature of the completion of ice crystallization during rewarming, the tailing in Bragg peaks disappeared, which indicated the formation of large and stable ice crystals, as were observed by X-ray CT.

Finally, the size and distribution of the ice grains formed in Sephadex^®^ beads were found to depend on the crosslink density.

## 5. Materials and Methods

### 5.1. Materials

Sephadex^®^ beads with different crosslink densities (i.e., G10, G15, G25, G50, and G100) were obtained from GE Healthcare UK Ltd. (Little Chalfont, Buckinghamshire, UK). The size of the beads’ diameter was 40–120 μm for G10, G15, and G100 and 50‒150 μm for G25 and G50, and the order of the density of the crosslinks is G10 > G15 > G25 > G50 > G100. The exclusion limits in *M*w/Da reported are 700, 1500, 5000, 30,000, and 100,000, and the amounts of water absorption in the swollen state (mL g^−1^_DM_) are 1.0, 1.5. 2.5, 5.0, and 10.0 for G10, G15, G25, G50, and G100, respectively [16]. In the compartment of G25, 250‒300 water molecules could be accommodated, assuming a spherical form [2]. The water content of the beads was adjusted to 50 wt % by the addition of distilled water, as the ice crystallization exotherm during rewarming in the DSC trace was most remarkably observed around the water content with a G25 gel.

### 5.2. X-ray CT Measurement

Sephadex^®^ G10, G25, and G50 were used in this experiment. G100 beads became too sticky, losing their bead structure when they were added with water, making it difficult to prepare G100 bead samples in quartz capillaries. So, G50 beads were used for the X-ray CT measurement instead of G100 beads as the bead sample with low crosslink densities (see the Discussion section). After having been dried at 60 °C for 20 h, each Sephadex^®^ bead sample containing 50 wt % water was prepared by the addition of distilled water to the dried beads. For the preparation of samples used in the X-ray CT measurement, the beads were densely packed by 2‒3 mm length in a quartz capillary with an outside diameter of 0.7 mm and about 10 mm length. Both ends of the capillary containing bead samples were then sealed with silicon grease to prevent the evaporation of water. Samples prepared were cooled from 20 to −40 °C at the rate of 1 °C min^−^^1^ by using a programmable freezer and kept below −40 °C until the measurement. In the case of G25, the refrozen samples re-cooled from the temperature of the completion of ice crystallization during rewarming (−11 to −5 °C) to −40 °C were also prepared. Cooling and re-cooling rates were 1 °C min^−1^, and the heating rate during rewarming was 5 °C min^−1^. Rapid freezing where samples were put into a deep freezer controlled at −85 °C prior to the measurement was also conducted. The cooling rate at that time was ca. 60 °C min^−1^.

**Figure 8:**
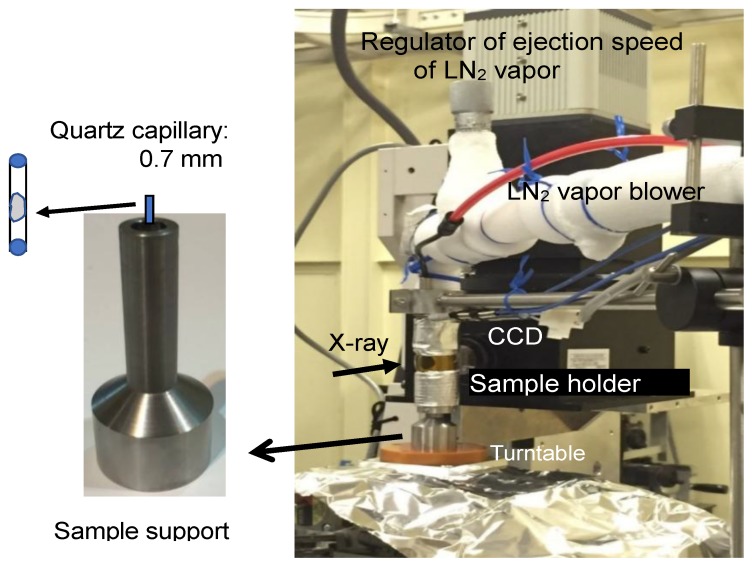
Photograph of an apparatus for X-ray CT measurement.

The X-ray CT measurement was carried out using the beam line of BL46XU at SPring-8 (Sayo, Hyogo, Japan). The X-ray wavelength was 0.1 nm, corresponding to 12.4 keV in energy. The angle of X-ray mirrors necessary to eliminate contamination of the X-ray by higher-order harmonics was set at 3.56 mrad. The size of the X-ray beam was 1 mm × 1 mm. The temperature of the frozen samples in the sample holder on the turntable (sample stage) was kept at about −40 °C by regulating the ejection speed of LN_2_ vapor introduced into the sample holder, and was measured with a thermocouple made of chromel-alumel. Frozen samples set on the turntable were continuously rotated 180 degrees at a rate of 1.2 degrees per second. The transmission images were acquired by using an X-ray imaging system composed of an AA50 X-ray imaging unit and a C4880-41S CCD camera (Hamamatsu Photonics K.K., Hamamatsu City, Shizuoka, Japan) set at the distance of 25 mm from the sample. A photograph of the apparatus for the X-ray CT measurement is shown in Figure 8. A set of 259 transmission images were acquired during the rotation. The exposure time for image acquisition was 250 msec. Two-dimensional images (tomograms) were reconstructed by the filtered back projection method. For each measurement, 1200 pieces of tomograms to be stacked were obtained. The size of pixels in the image data was 0.35 µm × 0.35 µm.

### 5.3. Observation by SEM

Samples for the observation by SEM were prepared in the same way as for the X-ray CT measurement. Sample beads of G10, G25, and G50 containing 50 wt % water were cooled to −40 °C at the rate of 1 °C min^−1^ by using a programmable freezer. After being freeze-dried, the beads were fixed on the aluminum stage with carbon tape and were sputter-coated with gold. Freeze-dried beads broken with a hammer were also prepared to observe the inside of the beads. SEM images were taken with a JSM-6010 PLUS_LA (JEOL Ltd., Akishima, Tokyo, Japan).

### 5.4. XRD Measurement

For the XRD measurement, Sephadex^®^ beads of G15, G25, and G100 were used without drying. Samples containing ca. 50 wt % water were put in cells with 5 mm inner diameter and 2 mm width, and were sealed with a pair of Kapton film windows of 25 μm in thickness. Samples were cooled from 25 to −40 °C at a rate of ca. 13 °C min^−1^, followed by stepwise heating to 25 °C with a temperature interval of 5 °C for G25 and G100, and 10 °C for G15. Annealing time at each temperature was about 5 min. Measurements of SAXS and WAXD were carried out using the beam line of BL40B2 at SPring-8. The X-ray wavelength was 0.1 nm, and the size of the X-ray beam was 0.1 mm × 0.1 mm. The range for scattering vector *q* (from the range for SAXS to that for WAXD) covered 0.04 < *q* (10 × nm^−1^) < 2.2, corresponding to 16‒0.28 nm of the real space. Camera length was 4153 cm for SAXS and 39 cm for WAXD. Exposure time for data acquisition was 60 s.
